# Cumulative Dietary Energy Intake Determines the Onset of Puberty in Female Rats

**DOI:** 10.1289/ehp.7039

**Published:** 2004-07-21

**Authors:** Jenny Odum, Helen Tinwell, Graham Tobin, John Ashby

**Affiliations:** ^1^Syngenta Central Toxicology Laboratory, Macclesfield, Cheshire, United Kingdom; ^2^Harlan Teklad UK, Bicester, Oxfordshire, United Kingdom

**Keywords:** energy intake, metabolizable energy, phytoestrogens, puberty, soy, uterotrophic assay

## Abstract

Laboratory animal diets for studies to determine the endocrine-disrupting potential of chemicals are under scrutiny because they can affect both assay control values and assay sensitivity. Although phytoestrogen content is important, we have previously shown that a phytoestrogen-rich diet and a phytoestrogen-free diet were equally uterotrophic to rats and advanced vaginal opening (VO) when compared with the standard diet RM1. Abolition of the effects by the gonadotrophin-releasing hormone antagonist Antarelix indicated that these effects were mediated through the hypothalamus–pituitary–reproductive organ axis. In the present study, we investigated the relationship between cumulative energy intake and sexual maturation in female rats. Infant formula (IF) at different concentrations and synthetic diets, with a wide range of metabolizable energy (ME) values, were used to modulate energy intake. Increasing energy intake was associated with an increase in uterine weight (absolute and adjusted for body weight) for both IF and the synthetic diets. In both cases, the increased uterine weight was directly proportional to energy intake. Body weight was unaffected by IF consumption but, in the case of the diets, was increased proportionally with energy consumption. Antarelix abolished the uterine weight increases with both formula and the diets, whereas body weight was unaffected. The mean day of VO was also advanced by high-ME diets and IF, whereas body weight at VO was unaffected. VO occurred at an energy intake of approximately 2,300 kJ/rat determined by measuring total food intake from weaning to VO, indicating that this cumulative energy intake was the trigger for puberty. ME is therefore a critical factor in the choice of diets for endocrine disruption studies.

The choice of laboratory animal diet for rodent studies to determine the endocrine-disrupting potential of chemicals is currently under intense scrutiny (Lawton 2003; Odum et al. 2001; Owens et al. 2003; Owens and Koëter 2003; Thigpen et al. 2003). This is because the diet selected can affect both assay control values and assay sensitivity; for example, uterine weight in control animals needs to be low to maximize the dynamic range of the uterotrophic assay. One contributing factor is the phytoestrogen content of the diet. Most of the commonly used laboratory animal diets are formulated with soy extracts, which contain the isoflavones genistein (GEN) and daidzein, and/or alfalfa (lucerne), which contains coumestrol (Patisaul and Whitten 1999). These phytoestrogens are estrogenic to rodents, causing effects such as increased uterine weight and advanced vaginal opening (VO) in immature animals, similar to effects observed with xenobiotic estrogens (Bickoff et al. 1962; Boettger-Tong et al. 1998; Casanova et al. 1999; Medlock et al. 1995; Thigpen et al. 1999; Tinwell et al. 2000; Whitten et al. 1992).

An analysis conducted as part of the recent Organisation for Economic Co-operation and Development (OECD) evaluation of the immature rat uterotrophic assay indicated that isoflavone levels greater than 325–350 mg GEN equivalents/kg diet should be avoided to maintain optimal assay sensitivity and dynamic range (Owens et al. 2003). The phytoestrogen content of diets is not, however, the only factor of importance. This is shown by our earlier demonstration that the phytoestrogen-rich diet Purina 5001 (Purina Mills, Inc., Richmond, IN, USA) and the phytoestrogen-free diet AIN-76A are equally uterotrophic to rodents, compared with the standard diet RM1, and that each is able to advance the mean day of VO in rats, again compared with RM1 (Odum et al. 2001). Further, we showed that coadministration of the gonadotrophin-releasing hormone (GnRH) antagonist Antarelix (ANT; Europeptides, Argenteuil, France) abolished the uterotrophic activity of both diets, indicating that these effects were mediated at the level of the hypothalamus to influence GnRH secretion (Odum et al. 2001). ANT is a synthetic peptide that was shown to be a GnRH antagonist in several animal models, including suppression of ovulation in rats and leutinizing hormone release in rams (Deghenghi et al. 1993). In a related series of experiments, we observed a correlation between the quantity of infant formula (IF) consumed by immature rats and mice and the magnitude of the resultant uterotrophic effect (Ashby et al. 2000). The uterotrophic effects were independent of the phytoestrogen content of the IF because they were abolished by inhibition of GnRH with ANT. In contrast, the uterotrophic effect of the reference synthetic estrogen diethylstilbestrol (DES) was unaffected by ANT (Ashby et al. 2000). These findings suggest that the type of food consumed by female rodents could influence the time of their puberty but that these influences were independent of phytoestrogen intake at the levels present in the foods used in these studies.

Energy intake is known to affect the onset of puberty in mammals; for example, pigs and rats with inadequate nutrition have retarded sexual development (Frisch et al. 1975; Kirkwood et al. 1987; Trentacoste et al. 2001). Energy balance in mammals is controlled by a series of complex central mechanisms that allow adaptive responses to situations of energy abundance or insufficiency. Two of the key hormones are leptin and ghrelin, which act as signals at either end of the spectrum (Zigman and Elmquist 2003). Leptin is secreted by adipocytes in response to increased food intake and energy balance. Its action on the brain and peripheral tissues results in activation of pathways suppressing food intake and increasing energy expenditure (Friedman and Halaas 1998). Ghrelin is released from endocrine cells in the stomach in response to decreased food intake and has the opposite effect to leptin (Gualillo et al. 2003). A definitive role for leptin in the onset of puberty has not yet been demonstrated (Ahima et al. 1977; Cunningham et al. 1999), but the importance of energy balance in sexual development led us to consider whether the effects described previously (Odum et al. 2001) were associated with the metabolizable energy (ME) of the diets/formulas evaluated and hence energy intake during the prepubertal period. However, the range of the ME densities of the diets used was small, and no useful correlation was found (Odum et al. 2001). Subsequently, Thigpen et al. (2002, 2003) evaluated several proprietary rodent diets containing phytoestrogens and with a wider range of ME densities. They observed a primary correlation of the phytoestrogen level of the diet, and a secondary correlation of the ME density of the diet, with the uterotrophic/VO activity of the diet to immature mice. However, food intake by the mice was not monitored, and this precluded accurate assessments of energy intake. Further, the analysis was complicated by studying concomitant differences in both dietary phytoestrogen levels and dietary ME values.

In the present experiments, we have investigated the relationship between total (cumulative) energy intake and sexual maturation in female rats. Two types of dietary modification were used. In one, IF (at different concentrations) and sugar solutions were used to modulate metabolic energy intake. In the second, open-formula synthetic phytoestrogen-free diets, with a wide range of metabolizable energies (8–22 kJ/g), were evaluated. Some experiments were conducted in the presence and absence of ANT to evaluate of the role of the hypothalamus–pituitary–reproductive organ axis on the effects observed.

## Materials and Methods

### Chemicals.

DES (> 99% pure), glucose, sucrose, and arachis oil (AO) were obtained from Sigma Chemical Co. (Poole, Dorset, UK). ANT was a gift from Europeptides, a Division of Asta Medica (Argenteuil, France). GEN was obtained from ChemService (West Chester, PA, USA). Halothane anesthetic was obtained from AstraZeneca (Alderley Park, Cheshire, UK).

### Animals.

Alpk:APfSD (Wistar-derived) rats, obtained from the AstraZeneca breeding unit (Alderley Park, Macclesfield, UK), were used in all studies. Studies were performed in accordance with the U.K. Animals (Scientific Procedures) Act (1986). Animal care and procedures were carried out according to in-house standards as described previously (Odum et al. 2001).

In the uterotrophic assays with IF and glucose, we used rats that were postnatal days (PND)21–22 on arrival into the laboratory (where birth is PND0). In the uterotrophic assays with the synthetic diets, we used weanling rats on PND18–19. This was because the former studies were carried out using the specifications described by Odum et al. (1997), and the latter studies followed the specifications required by the OECD evaluation of the uterotrophic assay (Kanno et al. 2003a, 2003b). Control uterine blotted weights for both series of studies were similar, generally between 20 and 30 mg. The sexual maturation study with IF was carried out in weanling rats on PND21–22 on arrival in the laboratory, whereas the study with the synthetic diets used weanling rats that were PND18–19 on arrival. To avoid confounding effects due to litter-mates or initial body weights, the weanling rats were taken from multiple litters and were randomly allocated to groups such that the initial group mean body weights were similar within experiments. In all experiments, animals were weaned on RM3 diet (Special Diet Services Ltd., Witham, Essex, UK) in the breeding unit and then fed the appropriate test diet upon arrival at the laboratory and for the duration of the assay. All solid diets, fluid diets, and drinking water solutions were available *ad libitum*.

### IF and sugar drinks.

IF (Infasoy; Cow and Gate, Trowbridge, Wiltshire, UK) was purchased from several outlets in Cheshire and Staffordshire (UK). It was prepared according to manufacturer instructions using sterile deionized water (considered as 100% strength throughout). The basic constituents are shown in Table 1. In one study, a dilute solution (33% recommended strength) and a more concentrated solution (200%) of IF were used. A glucose (6.6% wt/vol) solution in water was similarly prepared and evaluated. All drinking water solutions were prepared and replaced on a daily basis.

### Diets.

Two proprietary natural ingredient diets, Rat and Mouse No. 3 (RM3) and Rat and Mouse No. 1 (RM1) were supplied by Special Diet Services Ltd. (Witham, Essex, UK). RM1 has been consistently used as the standard diet in our postweaning studies since 1997 (Odum et al. 2001). A series of open-formula synthetic diets with a range of MEs (diets A–E) were produced by Harlan Teklad UK (Bicester, Oxfordshire, UK) and were based on AIN-76A (Knapka 1983). The constituents and proportions for all diets used, as well as the unique Harlan Teklad reference numbers for diets A–E, are listed in Table 1. AIN-76A and RM1 were included to provide links to our previous findings (Odum et al. 2001). In order to derive a wide range of ME densities, a base diet (designated diet B) was created. Diets with increasing ME densities were then achieved by substituting increasing proportions of lard for cellulose (diets C–E). A diet with an additional decrease in ME (diet A) was obtained by reducing the proportion of sucrose and maltodextrin. All diets were prepared as pellets.

We estimated ME densities of the synthetic diets using the values for protein, fat, and carbohydrate given by Blaxter (1989). The figure for casein protein takes into account the fact that it contains 10% moisture and 1% fat. The energy in the minerals and vitamins was derived from the excipients. Protein, vitamins, and fatty acids were maintained at a constant level in all the diets. The diets lowest in fat contained sufficient essential fatty acids to meet normal dietary requirements. The values for RM1 and IF were as reported by the manufacturer (Table 1). Total ME intake over the duration of the studies was calculated from solid and liquid food consumption data and the ME content of the diets and drinks.

### Analysis of diets for phytoestrogens.

We analyzed IF for daidzein and GEN content using the method described previously by Odum et al. (2001) and Owens et al. (2003). The limits of detection were 0.1 μg/g diet. The phytoestrogen aglycone contents of diet B (as representative of the phytoestrogen-free synthetic diets A–E) and RM1 were determined as described in detail by Wiseman et al. (2002). Portions of the diet (200 mg) were extracted by shaking with aqueous methanol at 60°C for 1 hr. The extracts were defatted with hexane and hydrolyzed to the aglycones with dilute hydrochloric acid. The aglycones were then extracted with ether. Daidzein, GEN, glycitein, and coumestrol were detected and quantified against reference samples by liquid chromatography coupled with mass spectroscopy. Data were adjusted for extraction efficiency. Quality control was determined by the concurrent analysis of a soy flour of known daidzein and GEN content, and results were < 9% different from those expected. The limit of detection was 0.05 μg/g diet for daidzein, GEN, and glycitein and 0.1 μg/g diet for coumestrol.

### Animal studies.

In all experiments, weanling rats were fed IF or synthetic diets upon arrival in the laboratory. Uterotrophic assays were based on the protocol described by Kanno et al. (2003a, 2003b) where the basic end point is uterine weight. In the sexual maturation studies, dietary modulation continued from weaning to postpuberty, and end points related to puberty (e.g., VO) were monitored. A scheme of the experiments and the hypotheses that they were designed to address are shown in Table 2.

#### Uterotrophic assays.

Uterotrophic assays were conducted using IF at different concentrations selected to provide a concentration-dependent response (experiments 1 and 2). In experiment 3, we administered a 6.6% glucose solution, either alone or in addition to coadministration of 5 mg/kg body weight GEN. In these experiments, the normal drinking water supply was replaced with either IF or glucose solutions. RM1 was provided as an optional solid food. Control rats were fed RM1 and water. Three uterotrophic assays were conducted using the pelleted synthetic diets (experiment 4 of 4 days’ duration and experiments 5 and 6 of 6 days’ duration). Control rats were fed RM1. Rats were housed up to five per cage. Food and fluid were available *ad libitum* and monitored (by cage) daily.

In experiments 1, 4, and 6, the GnRH antagonist ANT was coadministered at a dose of 300 μg/kg/day by subcutaneous (sc) injection (dosing volume, 1.5 mL/kg; Odum et al. 2001). In experiment 3, GEN was coadministered orally at 5 mg/kg/day in AO (dosing volume, 5 mL/kg). DES was used as a positive control in all studies. In experiments 1–3, DES was administered in the drinking water at either 10 or 20 μg/L, starting on the day of arrival of the rats at the laboratory and continuing throughout the experiment. In experiments 4–6, it was administered by sc injection at 5 μg/kg/day with a dosing volume of 1.5 mL/kg. Some animals received both DES and ANT or DES and AO, administered by two successive sc injections made within 5 min of each other. Rats administered DES were fed RM1. Control animals received vehicle only. Dosing of compounds (by sc or oral routes) commenced on the day after the rats had been placed on the test diets and continued daily. Animals were killed by an overdose of halothane 24 hr after the final chemical administration. Uteri were removed, blotted, and weighed as described previously (Odum et al. 1997).

#### Sexual maturation studies.

Weanling rats were provided with IF solutions, in place of drinking water supply, and RM1 diet in experiments 7 and 8. In experiment 9, weanling rats were fed either diet B or diet D instead of RM1. Control animals were fed RM1 in all experiments. Experiment 7 also contained a group of “heavy” control animals consisting of a group of the heaviest animals selected from the required weight range. Consequently, in the sexual maturation studies, the initial weights of the standard RM1 control group and the IF group were similar, whereas the weight of the “heavy control group” was greater. DES (30 μg/L in the drinking water) was administered in experiment 9 as a positive control with RM1 as the diet. This concentration of DES has previously been shown to decrease the mean age at VO by 7 days in the absence of changes in body weight (Odum et al. 2002). All diets and drinking water were available *ad libitum*. Rats in experiment 7 were housed singly, whereas rats in experiments 8 and 9 were housed in groups of five. Food and fluid consumption were monitored daily. VO was monitored daily from PND21, and individual body weights on the day of VO were recorded.

The age at first and second estrus were determined in experiment 8 by analysis of daily vaginal smears that were taken from the day of VO to PND65. First estrus was defined as the first day on which only cornified epithelial cells were observed on the vaginal smear. Second estrus was defined as the day on which a smear indicating estrus fell within a run of smears clearly showing the correct cyclic sequence of proestrus, estrus, metestrus, and diestrus.

Animals were killed on PND41, when all animals had open vaginas (experiments 7 and 9), or PND118, after second estrus (experiment 8). Liver, kidney, and uterine weights were determined at necropsy.

### Statistical methods.

For uterotrophic assays, we analyzed uterine weights by covariance with the terminal body weights. Terminal body weights were analyzed by covariance with initial body weights. Differences from control values (RM1 or RM1 with AO, as appropriate) were assessed statistically using a two-sided Student’s *t*-test based on the error mean square from the analysis of covariance (ANCOVA). Relationships between energy intake and body or uterine weight were analyzed by linear regression.

For sexual maturation studies, analysis of variance (ANOVA) was carried out on body weights, food consumption, and organ weights. Organ weights were also analyzed by covariance with the terminal body weights (Shirley 1996). VO was analyzed by Fisher’s exact test on the proportions of animals recorded each day with VO and by ANOVA for the observed days of VO and body weights at the time of VO. Differences from control values in all cases were assessed statistically using a two-sided Student’s *t*-test based on the error mean square from the ANOVA or ANCOVA. Analyses were carried out with SAS software (Version 8; SAS Institute, Inc., Cary, NC, USA).

## Results

### Diet analyses.

The synthetic diets A–E were free from daidzein, GEN, glycitein, and coumestrol. RM1 contained low levels of the phytoestrogens daidzein, GEN, and glycitein (11, 9, and 2 μg/g diet, respectively) and non-detectable levels of coumestrol. IF contained 45.7 μg daidzein and 133.4 μg GEN per gram dry formula (glycitein and coumestrol were not analyzed). ME values for the diets and IF are shown in Table 1.

### Uterotrophic assays.

**Figure 1:**
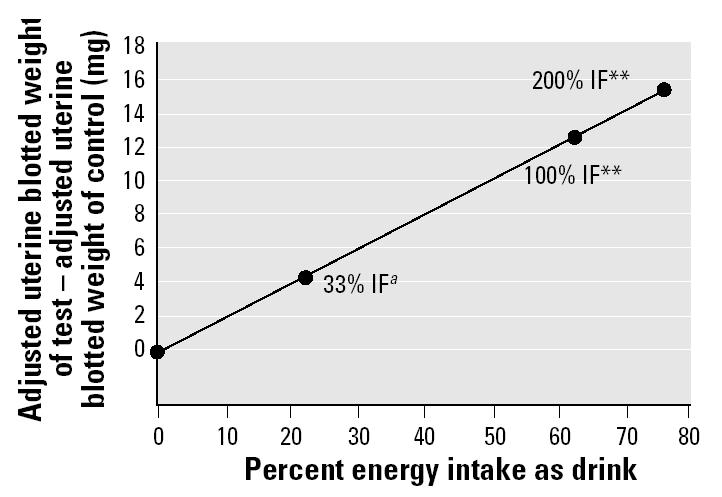
Total energy intake for rats drinking IF (33%, 100%, or 200% solutions) shown plotted against the increase in uterine weight above control levels (RM1 diet and water; all animals had access to RM1 diet). *R*^2^ = 0.99, *p* < 0.01. Data are based on experiment 2 (Tables 2 and 3).
***^a^***Uterine weight increase not significant. **Uterine weight increase significant at *p*< 0.01

**Figure 2:**
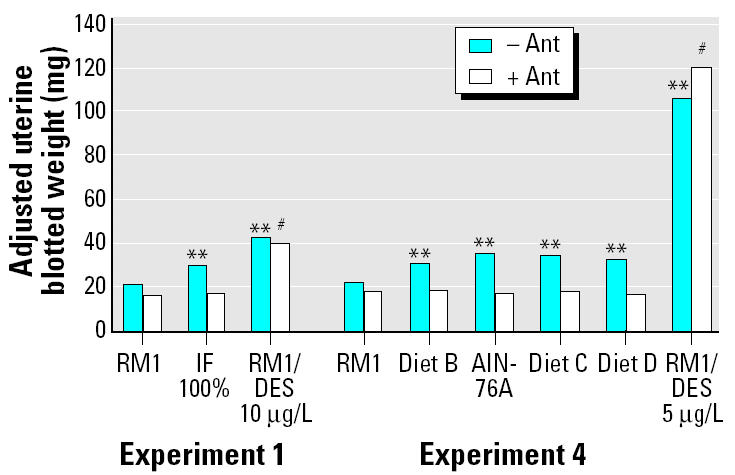
The effect of ANT (0.3 mg/kg/day, sc) on adjusted blotted uterine weights of rats fed IF or synthetic diets or dosed with DES [10 μg/L in drinking water (experiment 1) or 5 μg/kg/day sc (experiment 4)] in 4-day immature rat uterotrophic assays (Tables 3 and 4, respectively). Values are ANCOVA-adjusted means.
**p < 0.01 compared with RM1 control. ^#^*p* < 0.01 compared with RM1/ANT control.

In experiments 1 and 2 (Table 3), IF gave a positive uterotrophic response except when 33% IF was used (experiment 2, Table 3). All increases in uterine weight (compared with RM1 controls) occurred without significant effects on final body weights, except for the 200% IF group (Table 3). Energy intake was also increased above the RM1 controls in animals consuming IF. In experiment 2, uterine weight was increased proportionally with increasing IF concentration and energy intake (Figure 1). Coadministration of the GnRH antagonist ANT abolished the uterine weight increases induced by IF but did not affect the response given by DES (experiment 1, Table 3; Figure 2).

Administration of a solution of glucose to rats in 4-day uterotrophic assays (experiment 3, Table 3) had no effect on uterine weight. The concentrations were chosen based on the presence of 6.6% glucose in IF. Uterine weight was also unaffected by GEN at 5 mg/kg/day; this was the calculated daily intake of isoflavone in human infants consuming IF (at 100% concentration). The lack of effect is as expected from the dose response of GEN in the uterotrophic assay (Kanno et al. 2003a).

The results of the uterotrophic assays with the synthetic diets are shown in Tables 4 and 5. In all cases, uterine wet weight increased as energy intake increased in animals fed the synthetic diets. Body weights also increased, but uterine weights adjusted for covariance with terminal body weights were still increased.

**Figure 3:**
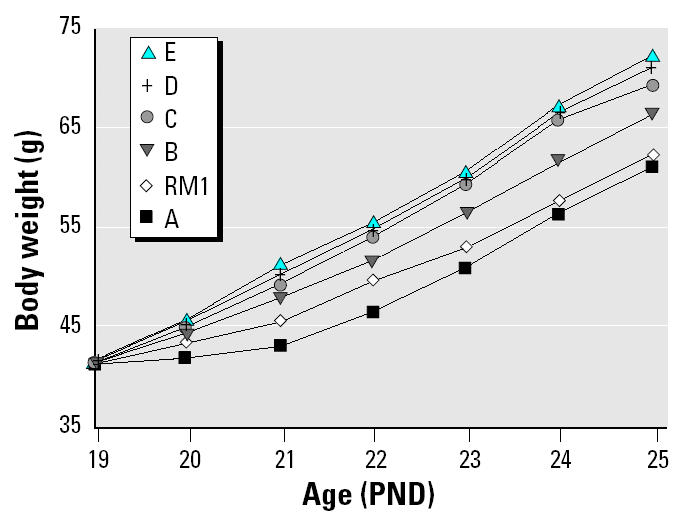
Body weights of female rats fed synthetic diets with different ME content in the uterotrophic assay (experiment 6, Table 5). For clarity, groups receiving ANT and/or DES are not shown. Statistically significant reductions (*p* < 0.01 compared with RM1 control) occurred with diet A on days 2 and 3 and increases occurred with diet B from day 4 onward and with diets C, D, and E from day 2 onward.

**Figure 4:**
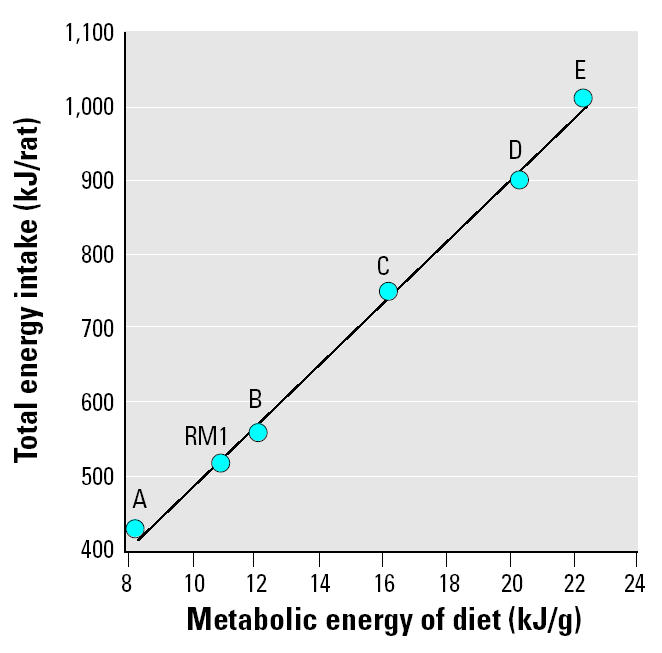
The relationship between total energy intake (kJ/rat) and the ME content (kJ/g) of RM1 and diets A–E between PND19 and PND25 for groups not receiving ANT in experiment 6 (Table 5).
*R*^2^ = 1.00, *p* < 0.01.

**Figure 5:**
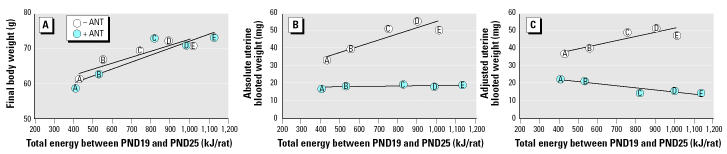
Final body weight (*A;* –ANT *R*^2^ = 0.86, *p* < 0.05; + ANT: *R*^2^ = 0.85, *p* < 0.05) and absolute (*B;* –ANT: *R*^2^ = 0.82, *p* < 0.05) and adjusted (*C;* –ANT: *R*^2^ = 0.78, *p* < 0.05) uterine weight plotted as a function of increasing total energy intake for animals fed diets A–E over 6 days (experiment 6, Table 5).

In experiment 4, rats were fed diets B–D and AIN-76A for 4 days, and the energy content of the synthetic diets ranged from 12.1 to 20.3 kJ/g. Absolute and adjusted uterine weight was significantly increased from 21 mg (RM1 control) to a maximum of approximately 35 mg by all the synthetic diets. The increase was abolished by coadministration of the GnRH antagonist ANT, when all diet groups attained absolute uterine weights of 17–19 mg (Table 4, Figure 2). Coadministration of ANT to the DES group had no effect on uterine weight (Figure 2). In experiment 5, the duration of the experiment was increased to 6 days in an attempt to enhance the sensitivity of the assay. Absolute and adjusted uterine weights for rats consuming diets B–D were significantly increased to a maximum of approximately 48 mg, and energy consumption was increased concomitantly (Table 5). In experiment 6, two more diets (diets A and E) were evaluated, expanding the ME range to 8.2–22.3 kJ/g. Diets were also fed for 6 days. The body weight curves (Figure 3) display a clear relationship between increasing energy intake and body weight, with body weights of animals fed diets B, C, D, and E being significantly increased relative to the RM1 control. Total energy intake over the 6 days was proportional to the ME of the diets (Figure 4). Coadministration of ANT had no effect on body weight (Table 5). Absolute and adjusted uterine weights were again significantly increased in animals fed the synthetic diets B–E compared with those fed RM1. In animals receiving diet A, with the lowest ME, absolute uterine weight was not significantly increased, but the increase in adjusted uterine weight was significant. A plateau was reached at absolute uterine weights of approximately 50–55 mg, suggesting that this may be the limit of prepubertal stimulation of uterine growth by manipulation of energy intake (Table 5). ANT again abolished the increases in uterine growth, reducing all absolute uterine weights to 16–19 mg (Table 5). The relationship between energy intake and either final body weight, absolute uterine weight, or uterine weight adjusted for body weight, for the data from experiment 6 (Table 5), was analyzed by linear regression (Figure 5).

In the uterotrophic assays with synthetic diets, the SDs for uterine weights were generally at least double those obtained with RM1. The reason for this is not clear. When ANT was coadministered, the SDs for uterine weight were smaller and less variable. We carried out an experiment in which rats were fed diet D under conditions of both single and group housing—in case competition for food within the cage was a factor—but SDs in both cases were similarly large (data not shown). We also attempted to reduce the uterine weight of rats fed diet D to that of rats fed diet B by restricting food (and therefore caloric) intake. The restriction achieved, however, was only partial because the animals ate their allocated amount of diet D so quickly that they would have been without food for long periods of the night. This was considered to be unacceptable for our animal license, and therefore the rats were given more food. A total energy intake of 889 kJ over 6 days was achieved in restricted animals fed diet D compared with 831 and 935 kJ for animals fed diets B and D, respectively, *ad libitum*. Uterine weights were 47.5 ± 14.4 mg in animals fed restricted amounts of diet D compared with 44.4 ± 9.3 and 51.0 ± 6.9 mg for animals fed diets B and D, respectively, *ad libitum*. This followed the trend established in Figure 4, but the reduction in uterine weight for the restricted animals was not statistically significant.

### Sexual maturation studies.

**Figure 6:**
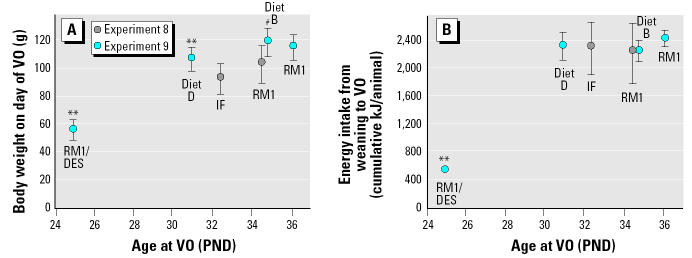
Body weights at VO (*A*) and cumulative energy consumption from weaning to the mean day of VO (*B*) for rats in the female sexual maturation studies (experiments 8 and 9, Tables 6 and 7). Values shown are mean ± SD.
**p < 0.01 for body weights at VO for diet D (and RM1/DES 30 μg/L drinking water) compared with RM1. ^#^*p* < 0.01 for body weights at VO for diet B compared with diet D; there were no statistically significant differences in energy intake between diets RM1 and IF (experiment 8) or RM1 and diets B or D (experiment 9).

Rats consuming IF (at 100% concentration) achieved VO approximately 2 days earlier than those fed RM1 alone, whereas body weights at VO were lighter (experiment 7, Table 6) or unaffected (experiment 8, Table 6; Figure 6). The group of heavy control animals (experiment 7) was significantly heavier at VO than were the “standard” controls, but age at VO was not different. Age at first and second estrus was significantly reduced by approximately 2 days for rats consuming IF (experiment 8, Table 6). No differences in the length of the estrus cycle were observed between groups.

Animals fed the synthetic diets D and B had increased body weights (from PND19.5 and 28.5, respectively) when compared with RM1 controls (data not shown). Rats receiving RM1 plus DES had reduced body weights from PND33.5 forward (experiment 9, Table 7). Consumption of diet D was reduced from PND26.5 compared with that of the RM1 controls, whereas consumption of diet B was similar to that of controls (data not shown). Compared with the control group fed RM1, VO occurred 1.3 days earlier in rats fed diet B and 5.2 days earlier in rats fed diet D (both advances statistically significantly different from the RM1 values and from each other). Body weight at VO for animals fed diet B was not different from those fed RM1, but animals fed diet D were significantly lighter at VO (the difference between diets B and D was also statistically significant; Table 7). Cumulative energy intake at the age of VO gave a consumption of approximately 2,300 kJ/rat at the time of VO for each of the three diets (Table 7, Figure 6). There was no statistical difference in energy intake up to the age of VO across the three diets. The figure of approximately 2,300 kJ/rat to day of VO is similar to the values observed in the IF studies (Table 7, Figure 6).

DES treatment resulted in an 11.2 day advance in VO, and the body weight at VO and the energy intake up to the time of VO were dramatically reduced (Table 7). DES intake was calculated to be 5.3 μg/kg/day over the whole study.

Absolute and adjusted liver and kidney weights were increased in animals fed diets B and D. There were no changes in uterine weights with either synthetic diet (Table 8), nor were there any organ weight changes after DES treatment. No organ weight changes were observed in animals fed IF (data not shown). The increase in relative liver and kidney weights was observed for AIN-76A previously (Odum et al. 2001) and has no obvious explanation.

## Discussion

There is a current concern that the diets used in rodent endocrine toxicity studies may influence, either qualitatively or quantitatively, the outcomes of those studies (Ashby et al. 2000; Boettger-Tong et al. 1998; Casanova et al. 1999; Lawton 2003; Odum et al. 2001; Owens et al. 2003; Thigpen 1999, 2002, 2003; Tinwell et al. 2000). Most of the studies cited above have concentrated on the possible effects of dietary phytoestrogen on rodent sexual maturation, but some studies have also attempted to evaluate the possible effects of changes in the ME of the diets (Ashby et al. 2000; Odum et al. 2001; Thigpen et al. 2002; 2003; Tinwell et al. 2000). However, these attempts have been rendered opaque by the concomitant effects induced by dietary phytoestrogens, failure to monitor food intake (reliance being placed solely on the stated ME values of the diets), and the use of proprietary diets that provide only a narrow range of MEs. The present studies have overcome these problems by using synthetic diets devoid of phytoestrogens—but with a wide range of ME values—and by monitoring food intake, leading to accurate assessments of cumulative energy intake. The soy-based IF was similarly studied, after establishing that the levels of phytoestrogen present in it are below those that affect the sexual maturation end points evaluated. Our conclusions from this study are as follows.

Increasing the ME density of the synthetic diet increases body weight (Tables 4 and 5, Figure 3) and total energy intake (Tables 4 and 5, Figure 4) proportionally. Body weight is increased for diets A–E over 6 days (Figure 3) and is maintained until PND41 for the two diets evaluated over that period (diets B and D, Table 7). RM1 diet, which is substantially different in its makeup from diets A–E, produces a growth curve consistent with its ME being between that of diets A and B (Figure 3).

Faced with the choice between RM1 diet and IF solution, rats select the latter, the strength of the preference being in proportion to the strength of the IF solution (experiment 2, Table 3). As the IF intake increases, so also does the total energy intake of the animals (Table 3). Unlike with the diets, increased energy intake from drinking IF is not closely associated with an increase in body weight. Only in the case of the 200% IF solution did body weight increase significantly over 4 days (Table 3), and exposures over longer periods led to variable effects on body weight (Table 6).

Increasing energy intake is associated with an increase in uterine weight for both IF (Table 3) and the diets A–E (Tables 4 and 5). In the case of IF, the increase in uterine weight over the concurrent controls was directly proportional to the percentage of energy intake via drink (Figure 1). We have shown previously (Ashby et al. 2000) that the uterotrophic activity of the IF brand used in this study is shared by two other proprietary brands of soy-based IF. We have also shown that rats elect to drink much less of a cow’s milk–based formula than they drink soy-based formula (Ashby et al. 2000). Consequently, energy intake through the cows’ milk–based formula is low (about the same as 33% IF; Table 3), and only marginal activity was observed for it in the uterotrophic assay (Ashby et al. 2000). In the case of the synthetic diets, the increase in uterine weight was proportional to the ME of the diets and to the total energy intake during the experiment (Tables 4 and 5, Figure 5). The nonresearch diets RM1 and AIN-76A also gave increases in uterine weight proportional to their ME values and total energy intake (Tables 4 and 5). Unlike with IF, body weights of the animals on the synthetic diets increased proportionally to the ME of the diets (Tables 4 and 5, Figure 5).

The uterotrophic activities of IF and the diets were abolished by coadministration of the GnRH antagonist ANT, but the uterotrophic activity of DES was unaffected (Tables 3–5, Figure 2). This confirms that the uterotrophic activity of DES, and by analogy the dietary phytoestrogens studied by Thigpen et al. (2003), act directly on the uterus, whereas the uterotrophic activity of the present synthetic diets, and IF, is stimulated by their effects on the hypothalamus. The independence of the uterotrophic effects from changes in body weight, discussed above for IF, is shown by the data in Figure 5 to apply equally to the diets. Figure 5 establishes that body weight increases induced by the diets, themselves in proportion to the ME of the diet, are not affected by ANT, whereas the concomitant increases in absolute and adjusted uterine weights are abolished by it. There are, therefore, two discrete influences at work in this study: increases in energy intake usually lead to increases in body weight, and increases in energy intake always lead to increases in uterine weight. Body weight is not always a good indicator of energy balance (the difference between intake and expenditure). It may be influenced by differences in gut contents, particularly when the nature of the diets is different (e.g., dry matter digestibility), and by the nature of the body constituents (body fat has eight times the energy content of bone-free lean tissue per gram) (Armitage et al. 1983). Differences in protein:energy ratio, such as seen in the diets in this study, can significantly affect the relative deposition of body fat and lean tissue (Blaxter 1975).

The prepubescent increase in uterine weight, and the advance in puberty induced by IF, was instigated by the rats through their voluntary drinking of IF in preference to eating the RM1. When presented with a 6.6% glucose solution (equivalent to the glucose content of IF), they obtained only approximately 20% of their energy intake from this solution, and this was insufficient to increase uterine weight (Table 3). Likewise, when the glucose solution was supplemented with 5 mg/kg GEN (the dose of phytoestrogens ingested by human infants drinking 100% IF), uterine weights did not increase significantly (Table 3). Further, the estimated daidzein and GEN intake during the sexual maturation study of IF (Table 6) was approximately 14 mg/kg, levels that are inactive in uterotrophic assays (Farmakalidis et al. 1985; Kanno et al. 2003a). The uterotrophic activity of the IF solution is therefore independent of its sugar content or its constituent phytoestrogens.

An advance in the day of VO mirrors the increases in uterine weight observed for animals maintained on diets B and D (Table 7) from weaning to PND41. Similar advances in the day of VO, together with advances in the day of first and second estrus, are seen for animals exposed to 100% IF from weaning to PND41 (Table 6). The day of VO for the four energy sources evaluated (RM1, diets B and D, and IF) correlates better with total energy intake up to the day of VO than it does with body weight on the day of VO (Figure 6). Supporting the secondary role of body weight on the day of VO is the fact that preselected heavy control animals maintained on RM1 have the same day of VO as do normal-weight control animals maintained on RM1, yet they have significantly heavier body weights at the day of VO (experiment 7, Table 6).

At the simplest level, these combined data indicate that events associated with the onset of puberty in female rats can be accelerated by increasing energy intake, enabled either by the use of high-ME diets or by the animals electing to drink large quantities of the relatively low-energy IF. The two peripubertal events monitored were the premature growth of the uterus and the early onset of puberty (VO and first estrus). These effects may be mechanistically distinct. The increases in uterine weight induced (from ~ 20 mg to ~ 40 mg) consistently fall short of the maximum uterine weight achieved at puberty, or after pseudoprecocious puberty induced by DES acting directly on the uterus (~ 130 mg). This suggests that the food effects are caused by the hypothalamus maximizing the prepubescent release of estrogens, as opposed to initiating full puberty. Previous studies (Ashby et al. 2000) have shown that the IF-induced uterine weight increase is inhibited by the estrogen receptor antagonist fulvestrant. This endogenous release of estrogens may be from the ovaries, initiated by GnRH acting on the pituitary-gonad axis via follicle-stimulating hormone, or from the adrenal glands via hypothalamic corticotrophin-releasing hormone acting on the pituitary–adrenal axis (Harvey and Everett 2003). The inhibition of uterine growth by ANT suggests the former, consistent with the demonstration by Branham and Sheehan (1995) that ovariectomy or adrenalectomy of PND6 rats decreased uterine growth. However, the early onsets of VO and, in the case of IF, of first and second estrus provide clear evidence of an advance in full hypothalamic puberty subsequent to achievement of a cumulative energy intake of approximately 2,300 kJ/rat post-weaning. Administration of ANT to rats during this period results in a total block on puberty (Ashby et al. 2002), so by definition, it was not possible use ANT to prove the involvement of the hypothalamus in these energy-induced pubertal effects.

Although there will be small variations in the efficiency with which the different proportions of dietary fat and carbohydrate in diets A–E would be converted to body fat, it is probable that the amount of fat deposited for any level of energy intake from these diets would be similar (not determined here). Body fat is an accurate measure of energy balance because it integrates the varying and sometimes small daily differences in intake and expenditure of energy. It is likely, therefore, that body fat content, rather than energy intake per se, is the critical variable. However, body weight does not provide an invariable indicator of body fat because of differences in the proportions of individual components of body weight, such as gut fill, body fat, and lean tissue—factors that may vary with the amount and nature of the diet, feeding pattern, and environment.

Earlier studies have failed to demonstrate clearly the central involvement of cumulative energy intake of puberty because of the concomitant presence of biologically active doses of phytoestrogens in the foods (Odum et al. 2001; Thigpen et al. 2002, 2003). It is therefore important to be aware of both the phytoestrogen content of diets and dietary energy intake during rodent studies evaluating the endocrine activities of chemicals. The latter will involve knowledge of the ME of the diet and awareness of differences in food intake between test and control groups.

**Figure 7:**
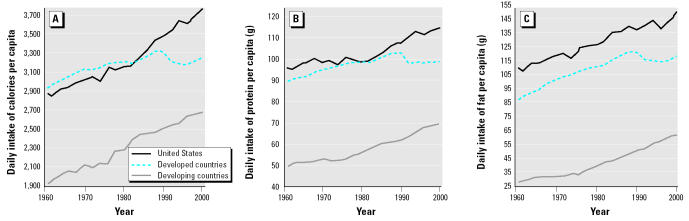
Intake of calories (*A*), protein (*B*), and fat (*C*) per capita from 1961 to 2000. Plotted using data from the database of the Food and Agricultural Organisation of the United Nations (FAO 2003).

The present observations relate directly to chemical safety assessments in rodents. However, the dramatic and continuing increase in human energy intake (Figure 7) indicates that the present observations may have a more general relevance to human health. For example, health breads such as Burgen (advertised as containing plant estrogens from soy and linseed; Allied Bakeries, Maidenhead, UK) show similar effects to those described here (Ashby and Tinwell 1998), and gross energy intake may also be associated with reports of a reduction in the age at which human females are entering puberty (Herman-Giddens et al. 1997). The present observations on IF are also relevant and are suggested to have the following implications for human infants drinking soy-based IF. First, the phytoestrogen content of these formulas leads to an average daily intake of approximately 4.5–10 mg/kg total phytoestrogens/day [Ministry of Agriculture, Fisheries and Food (MAFF) 1998; Setchell et al. 1997]. This level is known from other experiments to be devoid of reproductive effects in rodents (Lewis et al. 2003). Second, the observations made herein relate to the onset of puberty and are therefore of no relevance to infant humans exposed during the first few years of their life. Third, although the soy-based IF has the same ME as cows’ milk formulas, they may be more palatable to infants than cows’ milk formulas, and this may lead to excess energy intake, as happened in the present rodent studies. Anecdotal information indicates that mothers sometimes allow such excess intake to induce sleep. The only toxicologic implication for human infants is therefore one of possible excess calorific/energy intake.

## Figures and Tables

**Table 1:** Composition and ME content of the diets.

RM1*^a^*		IF (Infasoy)*^b^*		AIN-76A		Diets A–E (%)					
Constituent	g/100 g	Constituent	g/100 mL	Constituent	g/100 g	Constituent	A 02171*^c^*	B 01364	C 01365	D 02332	E 01366
Wheat/barley/wheat	88.5	Glucose syrup	NS	Casein	20	Casein	20	20	20	20	20
middlings		Carbohydrates	6.7	Sucrose	50	Sucrose	17.5	32.5	32.5	32.5	27.5
Soybean meal	6.0	Vegetable oils	NS	Corn starch	15	Maltodextrin	5	15	15	15	15
Whey powder	2.5	Fat	3.6	Cellulose	5	Cellulose	50	25	13.75	2.5	0
Soy oil	0.5	Soy protein isolate	NS	Corn oil	5	Lard	2.5	2.5	13.75	25	32.5
Minerals		Minerals	0.4	Minerals	3.5	Minerals	3.5	3.5	3.5	3.5	3.5
Vitamins	2.5	Vitamins		Vitamins	1	Vitamins	1	1	1	1	1
Amino acids				dl-Methionine	0.3	dl-Methionine	0.3	0.3	0.3	0.3	0.3
				Choline	0.2	Choline	0.2	0.2	0.2	0.2	0.2
				Ethoxyquin	0.001	Ethoxyquin	0.001	0.001	0.001	0.001	0.001
Total protein content (% wt/wt)	14.7	Total protein content (% wt/vol)	1.8	Total protein content (% wt/wt)	20	Total protein (% wt/wt)	20	20	20	20	20
Total ME (kJ/g diet)	10.9	Total ME (kJ/g diet)	2.8	Total ME*^d^* (kJ/g diet)	15.7	Total ME*^d^* (kJ/g diet)	8.2	12.1	16.2	20.3	22.3

a All values for RM1 are as stated on the manufacturer’s data sheet.

b Major constituents as stated on the Infasoy packaging; the quantities of glucose syrup, vegetable oils, and soy protein isolate were not specified (NS), but proportions of carbohydrates, fat, and protein were given.

c Unique Harlan Teklad reference numbers of the synthetic diets.

d ME was calculated using the following values (kJ/g constituent): casein, 16 kJ/g; sucrose, 16 kJ/g; corn starch, 16 kJ/g; maltodextrin, 16 kJ/g; cellulose, 0.3 kJ/g; corn oil, 37 kJ/g; lard, 37 kJ/g; minerals, 1.9 kJ/g; vitamins, 15.7 kJ/g; dl-methionine, 17 kJ/g; choline, 0 kJ/g; ethoxyquin, 0 kJ/g. The composition of the synthetic diets A–E was based on that of AIN-76A such that the protein content was identical but the carbohydrate and fat content were adjusted to give varying total ME values.

**Table 2:** Experimental scheme and hypotheses.

Experiment	Hypothesis	Treatment	Duration
Uterotrophic studies			
Experiment 1	IF consumption increases uterine weight	IF, ANT,*^a^* DES*^b^*	4 days (PND21–25)
	ANT antagonizes IF-induced uterine weight increase		
	ANT does not antagonize DES-induced uterine weight increase*^c^*		
Experiment 2	IF-induced uterine weight increase is dependent on IF concentration	IF (33–200%), DES	4 days (PND21–25)
Experiment 3	Glucose and GEN increase uterine weight	Glucose, GEN, DES	4 days (PND21–25)
Experiment 4	Consumption of synthetic diets with higher ME than RM1 increases uterine weight over 4 days	Synthetic diets, ANT, DES	4 days (PND18–22)
	ANT antagonizes synthetic diet-induced uterine weight increase		
	ANT does not antagonize DES-induced uterine weight increase		
Experiment 5	Consumption of synthetic diets with higher ME than RM1 gives greater uterine weight increase over 6 days	Synthetic diets, DES	6 days (PND18–24)
Experiment 6	Consumption of synthetic diets with low–high ME range shows correlation of ME with uterine weight	Synthetic diets, ANT, DES	6 days (PND18–24)
	ANT antagonizes synthetic diet-induced uterine weight increase		
	ANT does not antagonize DES-induced uterine weight increase		
Sexual maturation studies			
Experiment 7	IF consumption reduces age at VO	IF	20 days (PND21–41)
	Age-matched heavy controls have earlier VO		
Experiment 8	IF consumption reduces age at VO and age at first and second estrus	IF	97 days (PND21–118)
	Energy intake after weaning determines age at VO		
Experiment 9	Consumption of synthetic diets with higher ME than RM1 reduces age and body weight at VO	Synthetic diets, ANT, DES	23 days (PND18–41)
	Energy intake after weaning determines age at VO		
	DES treatment reduces age and body weight at VO*^d^*		
Experiment 9	Consumption of synthetic diets affects organ weight	Synthetic diets, DES	23 days (PND18–41)

a ANT is a GnRH antagonist used to determine whether GnRH mediates uterine weight increases.

b DES was used throughout as a positive control.

c As demonstrated previously (Ashby et al. 2000).

d As demonstrated previously (Odum et al. 2002).

**Table 3:** Immature rat uterotrophic assays with IF and sugar drinks (experiments 1–3).

Experiment/treatment	Total energy intake (kJ)*^a^*/rat	Percent energy intake as drink	Absolute uterine blotted weight (mg)*^b^*	Adjusted uterine blotted weight (mg)*^c^*	Final body weight (g)*^b^*	No.
Experiment 1						
RM1	238	0	20.7 ± 2.8	20.4	52.3 ± 4.5	10
RM1, IF 100%	416	88	29.9 ± 4.9**	29.8**	53.8 ± 3.3	10
RM1, DES 10 μg/L	243	0	41.8 ± 11.9**	41.6**	53.8 ± 4.1	10
RM1, ANT	237	0	16.3 ± 1.5	16.0	52.7 ± 4.1	10
RM1, IF 100%, ANT	431	89	16.9 ± 1.1	16.7	56.1 ± 3.7	10
RM1, DES 10 μg/L, ANT	254	0	39.7 ± 13.8*^#^*	39.5*^#^*	52.8 ± 4.4	10
Experiment 2						
RM1	311	0	27.3 ± 4.6	28.0	62.1 ± 5.4	9
RM1, IF 33%	341	22	31.5 ± 5.2	32.4	61.8 ± 5.8	10
RM1, IF 100%	464	62	40.7 ± 11.6**	40.5**	63.3 ± 5.2	10
RM1, IF 200%	547	75	44.7 ± 16.2**	43.3**	64.9 ± 5.3*	10
RM1, DES 10 μg/L	321	0	40.7 ± 6.0**	39.3**	65.0 ± 4.5	5
Experiment 3						
RM1, AO	222	0	22.2 ± 5.8	23.3	54.5 ± 6.4	9
RM1, glucose 6.6%	333	19.6	22.0 ± 6.0	22.7	55.2 ± 7.2	9
RM1, GEN 5 mg/kg/day	ND	0	21.3 ± 2.4	21.1	56.6 ± 7.4	9
RM1, glucose 6.6%, GEN 5 mg/kg/day	253	22.6	23.9 ± 5.9	24.8	54.8 ± 7.3	9
RM1, DES 20 μg/L	239	0	73.9 ± 15.3**	73.0**	57.8 ± 6.4	9

ND, not determined. DES was administered in drinking water.

a Total energy intake was calculated from the total amount of liquid and solid food consumed per rat over the duration of the study and their MEs. The ME value for RM1 was taken from the manufacturer’s data sheet; the ME value of IF was taken from information supplied by the manufacturer and adjusted for concentration where necessary; and the ME value of 16 kJ/g for glucose/sucrose was adjusted for concentration.

b Mean ± SD.

c Uterine weights adjusted for covariance with terminal body weights.

*p < 0.05 and

**p < 0.01 compared with RM1 or RM1/AO control.

# *p* < 0.01 compared with RM1/ANT control.

**Table 4:** Immature rat uterotrophic assays (4 days’ duration) using synthetic diets of different ME content (experiment 4).

Treatment	Diet ME intake (kJ/g diet)*^a^*	Total energy intake (kJ)*^b^*/rat	Absolute uterine blotted weight (mg)*^c^*	Adjusted uterine blotted weight (mg)*^d^*	Final body weight (g)*^c^*	No.
RM1/AO	10.9	222	21.4 ± 3.2	22.4	51.2 ± 7.2	10
Diet B/AO	12.1	243	29.2 ± 7.4**	30.7**	50.0 ± 8.0	10
AIN-76A/AO	15.7	325	35.8 ± 6.4**	34.9**	55.8 ± 8.0**	10
Diet C/AO	16.2	316	34.7 ± 9.1**	34.5**	54.0 ± 7.8**	10
Diet D/AO	20.3	218	34.4 ± 5.6**	32.5**	58.2 ± 7.6**	10
RM1/DES 5 μg/kg	10.9	434	105.1 ± 3.4**	106.1**	51.3 ± 6.6	4
RM1/ANT	10.9	222	17.1 ± 1.9	18.5	50.0 ± 6.8	10
Diet B/ANT	12.1	227	17.3 ± 2.4	18.8	49.8 ± 7.8	10
AIN-76A/ANT	15.7	336	17.8 ± 1.2	17.0	55.6 ± 6.4*^#^*	10
Diet C/ANT	16.2	314	18.1 ± 2.0	18.3	53.2 ± 8.3*^#^*	10
Diet D/ANT	20.3	422	18.8 ± 2.2	16.9	58.3 ± 6.5*^#^*	10
RM1/DES 5 μg/kg/ANT	10.9	232	119.2 ± 7.3*^#^*	120.6*^#^*	49.6 ± 7.5	4

ND, not determined. DES was administered sc.

a The ME value for RM1 was taken from the manufacturer’s data sheet.

b Total energy intake was calculated as the product of the total amount of food consumed per rat over the duration of the study and the ME of the diet.

c Mean ± SD.

d Uterine weights adjusted for covariance with terminal body weights.

**p < 0.01 compared with RM1 or RM1/AO control.

# *p* < 0.01 compared with RM1/ANT control.

**Table 5:** Immature rat uterotrophic assays (6 days’ duration) using synthetic diets of different ME content (experiments 5 and 6).

Experiment/treatment	Diet ME intake (kJ/g diet)*^a^*	Total energy intake (kJ)*^b^*/rat	Absolute uterine blotted weight (mg)*^c^*	Adjusted uterine blotted weight (mg)*^d^*	Final body weight (g)*^c^*	No.
Experiment 5						
RM1	10.9	485	27.6 ± 3.1	31.0	57.7 ± 6.5	10
Diet B	12.1	483	36.6 ± 6.7*	40.2**	57.5 ± 5.1	10
AIN-76A	15.7	696	45.0 ± 12.4**	42.1**	66.4 ± 5.1**	10
Diet C	16.2	666	44.7 ± 7.2**	42.9**	64.9 ± 5.8**	10
Diet D	20.3	907	47.5 ± 8.3**	42.0**	70.0 ± 6.4**	10
RM1/AO	10.9	471	30.4 ± 4.0	38.1	51.8 ± 8.2**	10
RM1/DES 5 μg/kg	10.9	530	131.2 ± 18.0**	133.9**	60.3 ± 5.6**	4
Experiment 6						
RM1/AO	10.9	520	26.4 ± 5.3	29.3	62.3 ± 5.4	10
Diet A/AO	8.2	426	33.2 ± 6.7	36.9*	61.0 ± 6.8	10
Diet B/AO	12.1	555	39.7 ± 7.8**	39.8**	66.5 ± 7.0*	10
Diet C/AO	16.2	897	50.8 ± 16.2**	49.0**	69.3 ± 5.0**	10
Diet D/AO	20.3	1,010	55.6 ± 18.6**	51.9**	72.2 ± 5.5**	10
Diet E/AO	22.3	481	50.9 ± 15.4**	48.0**	71.0 ± 4.1**	10
RM1/DES 5 μg/kg	10.9	434	122.4 ± 17.2**	124.6**	60.7 ± 6.3	4
RM1/ANT	10.9	493	16.1 ± 1.2	20.1	60.6 ± 4.6	10
Diet A/ANT	8.2	408	17.4 ± 1.5	22.6	58.9 ± 5.6*^#^*	10
Diet B/ANT	12.1	816	18.6 ± 2.1	21.5	62.3 ± 8.6	10
Diet C/ANT	16.2	314	19.2 ± 1.5	14.9	72.9 ± 6.3*^#^*	10
Diet D/ANT	20.3	991	18.4 ± 1.9	15.6	70.9 ± 5.1*^#^*	10
Diet E/ANT	22.3	1,131	19.1 ± 2.6	14.7	73.1 ± 6.9*^#^*	10
RM1/DES 5 μg/kg/ANT	10.9	465	153 ± 20.8*^#^*	154.5*^#^*	58.7 ± 2.7	4

DES was administered subcutaneously.

a The ME value for RM1 was taken from the manufacturer’s data sheet.

b Total energy intake was calculated as the product of the total amount of food consumed per rat over the duration of the study and the ME of the diet.

c Mean ± SD.

d Uterine weights adjusted for covariance with terminal body weights.

*p < 0.05 and

**p < 0.01 compared with RM1 or RM1/AO control.

# *p* < 0.01 compared with RM1/ANT control.

**Table 6:** Female sexual maturation of rats given IF (experiments 7 and 8).

						First estrus		Second estrus		
Experiment/treatment	Body weight at PND21 (g)	Body weight at PND41 (g)	Cumulative energy intake at VO*^a^* (KJ)	Age at VO (PND)	Body weight at VO (g)	Age (PND)	Weight (g)	Age (PND)	Weight (g)	No.
Experiment 7										
RM1	48.0 ± 5.6	145.1 ± 15.5	ND	33.7 ± 1.9	111.3 ± 8.5	ND	ND	ND	ND	10
RM1 heavy control	56.5 ± 1.7*	159.5 ± 13.2*	ND	33.3 ± 1.5	124.8 ± 12.9*	ND	ND	ND	ND	10
RM1/IF 100%	48.0 ± 5.6	164.6 ± 11.8**	ND	31.1 ± 1.5**	99.2 ± 12.4*	ND	ND	ND	ND	10
Experiment 8										
RM1	37.1 ± 5.7	140.7 ± 11.6	2,181 ± 425	34.5 ± 2.0	102.4 ± 13.2	37.1 ± 3.9	115.2 ± 19.8	44.6 ± 5.9	149.6 ± 23.0	45
RM1/IF 100%	37.5 ± 5.7	143.2 ± 14.2	2,249 ± 368	32.4 ± 1.2**	91.5 ± 10.8	35.3 ± 2.7*	108.3 ± 18.7	42.8 ± 5.4*	148.6 ± 28.4	61

ND, not determined. Values shown are mean ± SD.

a Cumulative energy intake was calculated from the amount of IF and food (and their MEs) consumed per rat up to VO.

*p < 0.05 and

**p < 0.01 compared with RM1 control; there were no statistically significant differences in energy intake at VO between RM1 and IF (experiment 8).

**Table 7:** Female sexual maturation of rats fed synthetic diets (experiment 9).

Treatment	Body weight at PND21 (g)	Body weight at PND41 (g)	Cumulative energy intake at VO*^a^* (kJ)	Age at VO (PND)	Body weight at VO (g)	No.
RM1	40.9 ± 4.0	137.5 ± 11.9	2,404 ± 108	36.1 ± 1.7	114.2 ± 9.1	20
Diet B	38.3 ± 4.2	147.8 ± 5.4**	2,214 ± 151	34.8 ± 1.5*	117.2 ± 9.9	20
Diet D	43.5 ± 4.1**	166.1 ± 10.6**	2,281 ± 208	30.9 ± 1.0**,#	105.1 ± 8.5**, #	20
RM1/DES 30 μg/L	39.7 ± 3.0	127.1 ± 12.8**	479 ± 58**	24.9 ± 0.7**	55.7 ± 7.4**	10

Values are mean ± SD. DES was administered in the drinking water.

a Total energy intake was calculated from the amount of food (and the MEs of the diets) consumed per rat up to VO.

*p < 0.05,

**p < 0.01 compared with RM1 control.

# *p* < 0.01 for age and body weight at VO for diets D and B; there were no statistically significant differences in energy intake at VO between RM1 and diets B and D when either RM1 or diet B was used as the control.

**Table 8:** Organ weights of female rats (at PND41) fed synthetic diets (experiment 9).

Treatment	Liver (g)	Kidney (g)	Uterus (mg)	No.
RM1				
Absolute	6.7 ± 0.7	1.2 ± 0.1	177 ± 43	20
Adjusted	7.2	1.3	178	
Diet B				
Absolute	8.2 ± 0.6**	1.8 ± 0.2**	203 ± 62	20
Adjusted	8.3**	1.8**	203	
Diet D				
Absolute	9.2 ± 0.9**	1.8 ± 0.2**	205 ± 43	20
Adjusted	8.5**	1.7**	203	
RM1/DES (30 μg/L)				
Absolute	5.8 ± 1.0	1.1 ± 0.1	188 ± 52	10
Adjusted	6.9	1.29	180	

Values shown are mean ± SD. DES was administered in the drinking water.

a Organ weights adjusted for covariance with terminal body weights.

**p < 0.01 compared with RM1 control.
